# Two Distinct Channels Mediated by m2mAChR and α9nAChR Co-Exist in Type II Vestibular Hair Cells of Guinea Pig

**DOI:** 10.3390/ijms14058818

**Published:** 2013-04-24

**Authors:** Tao Zhou, Yi Wang, Chang-Kai Guo, Wen-Juan Zhang, Hong Yu, Kun Zhang, Wei-Jia Kong

**Affiliations:** 1 Department of Otorhinolaryngology, Union Hospital, Tongji Medical College, Huazhong University of Science and Technology, 1277 Jiefang Avenue, Wuhan 430022, China; E-Mails: zhoutao2012ent@yahoo.cn (T.Z.); wangyi821@yahoo.cn (Y.W.); ckguo2255@sina.com (C.-K.G.); juan_364@163.com (W.-J.Z.); yuhong_0706@163.com (H.Y.); kkwd_010@163.com (K.Z.); 2 Institute of Otorhinolaryngology, Union Hospital, Tongji Medical College, Huazhong University of Science and Technology, 1277 Jiefang Avenue, Wuhan 430022, China; 3 Key Laboratory of Neurological Disorders of Education Ministry, Tongji Medical College, Huazhong University of Science and Technology, Wuhan 430022, China

**Keywords:** calcium-dependent potassium channel, muscarinic ACh receptor, nicotinic ACh receptor, vestibular hair cell

## Abstract

Acetylcholine (ACh) is the principal vestibular efferent neurotransmitter among mammalians. Pharmacologic studies prove that ACh activates a small conductance Ca^2+^-activated K^+^ channels (KCa) current (SK2), mediated by α9-containing nicotinic ACh receptor (α9nAChR) in mammalian type II vestibular hair cells (VHCs II). However, our studies demonstrate that the m2 muscarinic ACh receptor (m2mAChR) mediates a big conductance KCa current (BK) in VHCs II. To better elucidate the correlation between these two distinct channels in VHCs II of guinea pig, this study was designed to verify whether these two channels and their corresponding AChR subtypes co-exist in the same VHCs II by whole-cell patch clamp recordings. We found that m2mAChR sensitive BK currents were activated in VHCs II isolated by collagenase IA, while α9nAChR sensitive SK2 currents were activated in VHCs II isolated by trypsin. Interestingly, after exposing the patched cells isolated by trypsin to collagenase IA for 3 min, the α9nAChR sensitive SK2 current was abolished, while m2mAChR-sensitive BK current was activated. Therefore, our findings provide evidence that the two distinct channels and their corresponding AChR subtypes may co-exist in the same VHCs II, and the alternative presence of these two ACh receptors-sensitive currents depended on isolating preparation with different enzymes.

## 1. Introduction

Hair cells in a higher vertebrate’s vestibule are generally divided into two categories: the flask shaped type I vestibular hair cells (VHCs I) and the cylindrical shaped type II vestibular hair cells (VHCs II) [1]. VHCs II are innervated by a number of button-shaped efferent nerve endings, which are cholinergic efferent fibers demonstrated by investigation of ACh-esterase activities in rat [2] and studies of choline acetyltransferase (ChAT) activities in rat [2,3], human [4] and mouse [5]. All these results support that acetylcholine (ACh) is the principal inhibitory neurotransmitter mediating the efferent activities to the vestibule [6].

Cholinergic neurotransmission is mediated by two classes of ACh receptors (AChRs): ionotropic nicotinic AChRs (nAChRs), which are nonselective cation channels, and G-protein-coupled muscarinic AChRs (mAChRs), which act through second-messenger systems [7,8]. The differential expressions of α2–7, α9α10, β2–4nAChR subunits [9] and m1-m5mAChR subtypes [10] have been observed in the vestibular end organs of rat. Besides, m1, m2, m5mAChR subtypes have also been observed in human vestibular periphery by reverse transcription polymerase chain reaction (RT-PCR) studies [10,11]. Recently, immunohistochemical studies have further supported that m1-m5 mAChR subtypes were located in isolated VHCs II of pigeons [12], and RT-PCR has also provided strong evidence that m1-m5 mAChR subtypes were detected in isolated VHCs II of guinea pig [13].

Previous pharmacologic studies have proved that ACh activated a small conductance Ca^2+^-activated K^+^ (KCa) current (SK2) in mammalian VHCs II and outer hair cells (OHCs), which was mediated by the well-known α9nAChR and sensitive to apamin and strychnine [14–16]. However, our recent studies showed that ACh activated a big conductance KCa (BK) current in VHCs II of guinea pig, which was mediated by m2mAChR and sensitive to iberiotoxin (IBTX) and methoctramine [17–19], yet insensitive to strychnine and apamin. These two discrepant results implied that m2mAChR mediated BK currents and α9nAChR mediated SK2 currents did not appear at the same time, which also indicated that the two distinct channels mediated by m2mAChR and α9nAChR may exist in different VHCs II.

We found that collagenase IA was conventionally used to dissociate VHCs II in our studies [17–19], while most of the other researchers usually isolated mammalian VHCs II and OHCs by trypsin or collagenase IV [20,21]. So it was speculated that α9nAChR/SK2 complex might undergo proteolysis during the collagenase IA isolation process. Indeed, the enzymes could alter physiological responses of α9nAChR/SK2 complex. It has been reported that α9nAChR sensitive SK2 current was well preserved in frog saccular hair cells isolated by collagenase IV or trypsin, but this current in trypsinized cells could be totally abolished after brief exposure to protease VIII, protease XXIV or papain [16]. Moreover, Armstrong *et al.* had also showed that the electrical properties of frog saccular hair cells could be distorted by papain dissociation resulting in the absence of SK2 channels and the emergence of non-inactivating BK channels [22,23]. These results indicated that BK channel and SK2 channel may co-exist in the same saccular hair cells of frog.

Based on the above findings, we propose a hypothesis that m2mAChR and α9nAChR may co-exist in the same VHCs II and mediate distinct channels respectively, while these differential cholinergic responses depend on the isolating preparation with different enzymes.

Therefore, the aim of the present study was to explore the correlation between m2mAChR mediated BK currents and α9nAChR mediated SK2 currents in the same VHCs II of guinea pig. First, under the consistent experimental condition, we should demonstrate that these two cholinergic receptors α9nAChR and m2mAChR mediated two distinct channels in VHCs II of guinea pig isolated with trypsin or collagenase IA respectively. Then, we should further prove that α9nAChR and m2mAChR may co-exist in the same cell. Therefore, we designed a three-step experiment by whole cell patch-clamp recordings. Consequently we presented our results in three parts: (1) VHCs II isolated by collagenase IA routinely displayed the m2mAChR sensitive BK current; (2) VHCs II isolated by trypsin routinely displayed the α9nAChR sensitive SK2 current; (3) After brief exposing the patched cells isolated by trypsin to collagenase IA for 3 min, the α9nAChR sensitive SK2 current was abolished, while a non-inactivating BK current was activated by ACh. These findings provided evidences that the two distinct channels (BK and SK2) as well as the two ACh receptors (α9nAChR and m2mAChR) may co-exist in the same mammalian VHCs II, and the alternative presence of these two ACh receptors sensitive currents was dependent on the isolating preparation with different enzymes.

## 2. Results and Discussion

### 2.1. m2mAChR Sensitive BK Currents Exist in VHCs II Isolated by Collagenase IA

**Figure 1:**
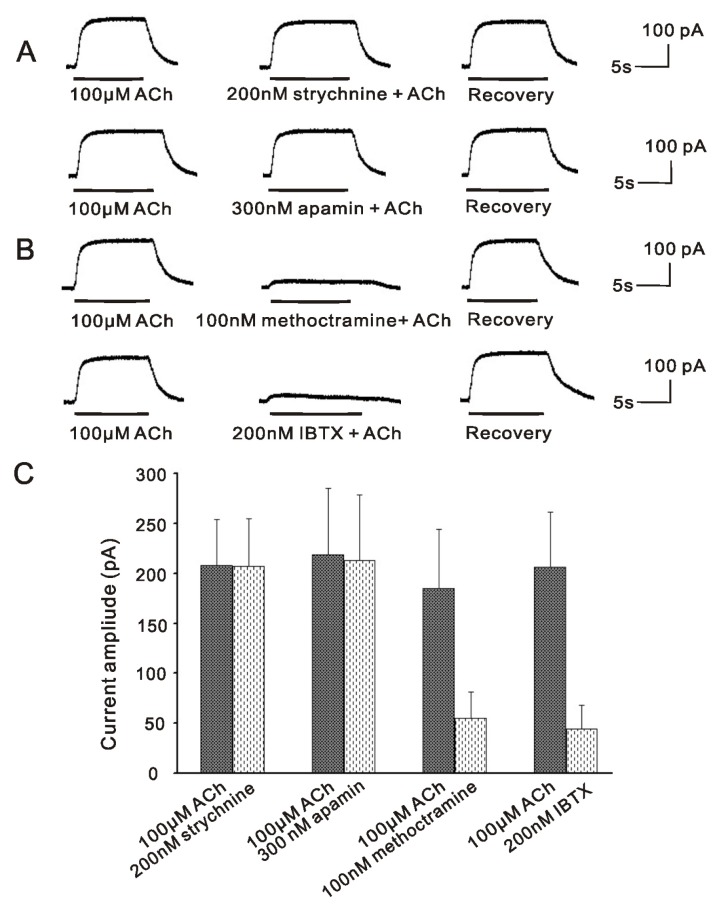
ACh-activated BK currents in collagenase IA isolated VHCs II. ACh routinely activated a non-inactivating outward current. (**A**) The current activated by 100 μM ACh was insensitive to strychnine and apamin, respectively; (**B**) The current activated by 100 μM ACh was potently inhibited by methoctramine and IBTX, respectively. All of these four results were obtained from the one same cell; (**C**) Bar histogram showed the effects of 200 nM strychnine, 300 nM apamin, 100 nM methoctramine and 200 nM IBTX on the current evoked by 100 μM ACh. Each point represents the mean ± SD of 5–6 cells. (V_hold_ = −50 mV).

Our previous finding showed that the BK currents activated by ACh in VHCs II isolated by collagenase IA were mediated by m2mAChR [19], which could be potently inhibited by the BK channel blocker IBTX [17,19] and m2mAChR antagonist methoctramine [19], but was insensitive to the SK2 channel blocker apamin [17] and α9nAChR antagonist strychnine [19]. In order to further confirm these results in the same cell under the consistent experimental condition, we again demonstrated the properties of currents activated by ACh in the same VHCs II isolated by collagenase IA. As shown in Figure 1, the outward currents activated by ACh (100 μM) were strongly inhibited by 200 nM IBTX and 100 nM methoctramine to 20.3% ± 7.1% (control, 206.4 ± 55.2 pA; ACh + IBTX, 44 ± 24.5 pA; *p* < 0.001; *n* = 6) and 28.6 ± 6.8% (control, 185.2 ± 59 pA; ACh + methoctramine, 55 ± 26.1 pA; *p* = 0.002; *n* = 6) respectively, but insensitive to 200 nM strychnine (control, 207.6 ± 46.3 pA, strychnine + ACh, 206.8 ± 47.8 pA, *p* = 0.981; *n* = 6) and 300 nM apamin (control, 218.3 ± 66.5 pA, ACh + apamin, 213 ± 65.4 pA, *p* = 0.914; *n* = 6). Therefore, these results showed that ACh activated a non-inactivating outward current in VHCs II isolated by collagenase IA, which was BK current and mediated by m2mAChR. As described in our previous findings [17,19], the SK2 channels and the α9nAChR were not activated at the same time.

### 2.2. α9nAChR Sensitive SK2 Currents Exist in VHCs II Isolated by Trypsin

**Figure 2:**
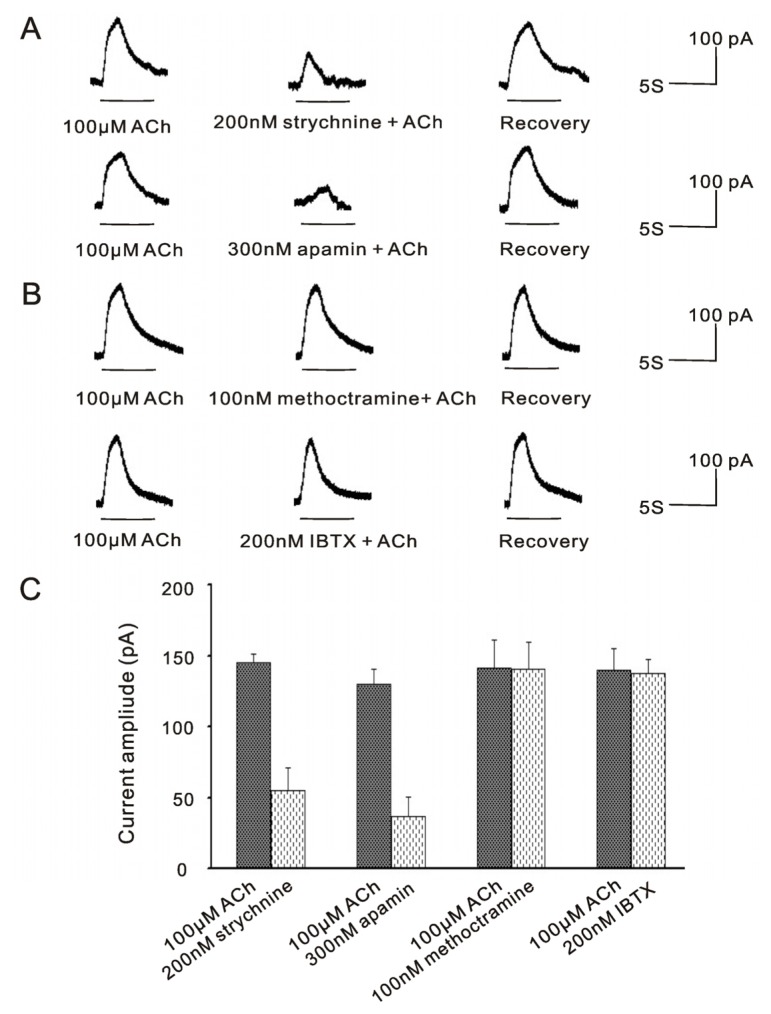
ACh activated SK2 currents in trypsin-isolated VHCs II. ACh routinely activated a fast inactivating outward current. (**A**) The current activated by 100 μM ACh was potently inhibited by strychnine and apamin, respectively; (**B**) The current activated by 100 μM ACh was insensitive to methoctramine and IBTX, respectively. All of these four results were obtained from the one same cell; (**C**) Bar histogram showed the inhibition effects of 200 nM strychnine, 300 nM apamin, 100 nM methoctramine and 200 nM IBTX on the current evoked by 100 μM ACh. Each point represents the mean ± SD of 6 cells. (V_hold_ = −50 mV).

It has been reported that α9nAChR sensitive SK2 currents were well preserved in frog saccular hair cells isolated by trypsin [16], which were sensitive to apamin and strychnine [14–16]. Here, we studied the properties of currents activated by ACh in VHCs II isolated by trypsin. As shown in Figure 2, the outward currents activated by 100 μM ACh in one same cell were potently inhibited to 37.6% ± 10.5% and 28.4% ± 10.6% by 200 nM strychnine (control, 145.3 ± 6.2 pA; ACh + strychnine, 54.8 ± 16.5 pA; *p* < 0.001; *n* = 6) and 300 nM apamin (control, 129.8 ± 10.5 pA; ACh + apamin, 36.8 ± 13.4 pA; *p* < 0.001; *n* = 6), respectively, but insensitive to 100 nM methoctramine (control, 141.3 ± 20 pA; ACh + methoctramine, 140.3 ± 19.5 pA; *p* = 0.945; *n* = 6) and 200 nM IBTX (control, 140 ± 14.7 pA; ACh + IBTX, 137.5 ± 10.1 pA; *p* = 0.789; *n* = 6). Therefore, these results showed that ACh activated a fast inactivating current, which was SK2 current and mediated by the α9nAChR in VHCs II isolated by trypsin. Meanwhile, the BK channels and the m2mAChR were not activated at the same time.

### 2.3. m2mAChR Sensitive BK Currents and α9nAChR Sensitive SK2 Currents Co-Exist in VHCs II

The above results indicated that there presented two distinct ACh receptors activated currents in VHCs II isolated by different enzymes. We noted that certain enzymes could affect or even damage the physiological properties of hair cells [16,22,23]. Therefore, we investigated the proteolytic effect of collagenase IA on the trypsin-dissociated cells to verify the hypothesis that two distinct channels mediated by m2mAChR and α9nAChR co-exist in VHCs II.

**Figure 3:**
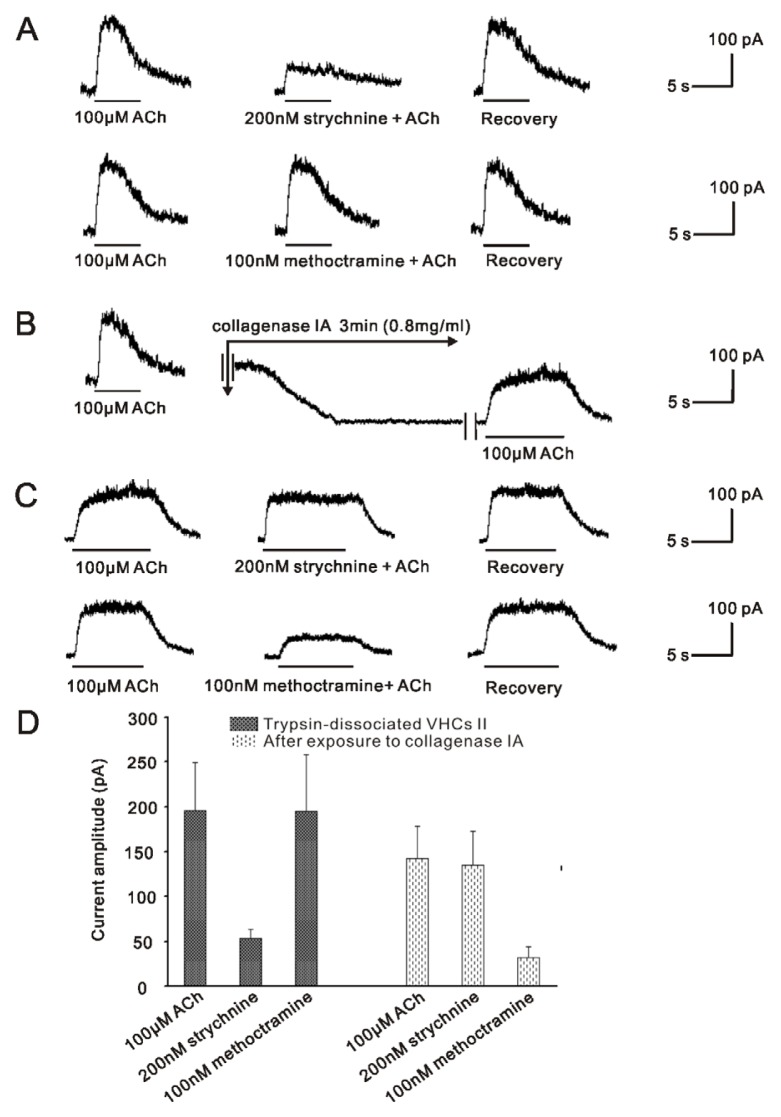
Change of the currents after exposure to collagenase IA on one trypsin-isolated cell. (**A**) Before exposure to collagenase IA, the trypsin-isolating cell displayed a typical fast inactivating current, which was strongly inhibited by 200 nM strychnine, but insensitive to 100 nM methoctramine; (**B**) 0.8 mg/mL collagenase IA dissociation solution was applied to the patched cell for 3 min. Note that there was a downward shift in the baseline; (**C**) After exposure to collagenase IA and incubated with normal external solution for 5 min, the fast inactivating current was totally abolished, while a non-inactivating current was activated. The non-inactivating current was insensitive to 200 nM strychnine, but could be potently inhibited by 100 nM methoctramine. All of these above results were obtained from one same cell; (**D**) Bar histogram showed the inhibition effects of 200 nM strychnine and 100 nM methoctramine on the current evoked by 100 μM ACh. Each point represents the mean ± SD of 6 cells. (V_hold_ = −50 mV).

As illustrated in Figure 3A, the fast inactivating currents activated by 100 μM ACh in one same trypsinized cell were dramatically reduced to 29.9% ± 5.8% by 200 nM strychnine (control, 196 ± 53 pA; ACh + strychnine, 53.6 ± 9.6 pA; *p* = 0.006; *n* = 6), while insensitive to 100 nM methoctramine (control, 196 ± 53 pA; ACh + methoctramine, 194.8 ± 63.4 pA; *p* = 0.936; *n* = 6). Next, 0.8 mg/mL collagenase IA dissociation solution was applied to this patched cell for 3 min. There was a downward shift in the baseline (Figure 3B). Then, this patched cell was incubated with the normal external solution for 5 min. Finally, 100 μM ACh was applied again. Interestingly, re-application of ACh activated a non-inactivating current, which was different from the previously recorded fast inactivating SK2 current. As shown in Figure 3C, this non-inactivating current was insensitive to 200 nM strychnine (control, 142.2 ± 36.4 pA; ACh + strychnine, 135.2 ± 37.5 pA; *p* = 0.883; *n* = 6), but could be potently inhibited by 100 nM methoctramine to 22.9% ± 6.7% (control, 142.2 ± 38.7 pA; ACh + methoctramine, 32 ± 12.5 pA; *p* = 0.002; *n* = 6). Similar results were obtained from six cells. These results not only showed that the α9nAChR responses were replaced by the m2mAChR responses after brief exposure to collagenase IA, but also suggested that these two distinct channels and their corresponding ACh receptors may co-exist in the same VHC II.

### 2.4. Discussion

In the present study, we verified the hypothesis that m2mAChR, α9nAChR and the two distinct channels (BK and SK2) may co-exist in the same VHCs II of guinea pig. We showed that m2mAChR sensitive BK currents were located in VHCs II isolated by collagenase IA, and α9nAChR sensitive SK2 currents were located in VHCs II isolated by trypsin. After brief exposing the patched cells isolated by trypsin to collagenase IA, α9nAChR sensitive SK2 currents were abolished, while m2mAChR sensitive BK currents were activated. These results suggested that these two distinct channels and their respective ACh receptors may co-exist in the same VHCs II of guinea pig, and the alternative presence of these two ACh receptors-sensitive currents was dependent on the isolating preparation with different enzymes.

#### 2.4.1. BK Channel and SK2 Channel Co-Exist in the Same VHCs II, but do Not Play Their Roles at the Same Time in Vestibular Efferent Inhibition as in OHCs

The inner ear comprises two main functional parts, cochlea and vestibule, which are responsible for sound perception and balance maintenance respectively. It has been established that ACh is the main efferent neurotransmitter in mammalian inner ear [6,24], which activates α9nAChR with the subsequent opening of SK2 channel [14–16]. In the mammalian vestibular neuroepithelium, VHCs II are very small and rare sensory cells, with which the patch clamp experiments are very hard to manipulate, so the detailed mechanisms underlying cholinergic efferent inhibition in vestibule remain unclear, except for the well known α9nAChR mediated SK2 channel [14–16]. However, there has been morphological evidence recently showing that another kind of calcium-activated potassium channel named BK channels are expressed in VHCs II of rat by immunohistochemical study [25]. Besides, our previous studies have also demonstrated that ACh activated BK currents are present in the VHCs II of guinea pig [17,18]. Further studies showed that this inhibition is mediated by m2mAChR [19]. In the present study, we again demonstrated that m2mAChR mediated BK channels in VHCs II isolated by collagenase IA, which was not activated by α9nAChR (Figure 1). All these results indicate that BK channel is also involved in cholinergic efferent inhibition in vestibule. Moreover, after brief exposing the patched cell isolated by trypsin to collagenase IA, α9nAChR sensitive SK2 currents were abolished, while m2mAChR sensitive BK currents were activated (Figure 3). These results imply that BK channel and SK2 channel may co-exist in the same VHCs II, so do their respective upstream receptors α9nAChR and m2mAChR.

Recently, in the OHC area of the adult rat cochlea, immunohistochemical studies have shown that BK-labeled plaques are observed beneath the nuclei of the OHCs except at the most apical cochlear region [26,27], and are located at the interface between presynaptic olivocochlear efferent terminals and the post-synaptic OHC membrane [26,28,29]. Since SK2 channel is almost expressed in all the apical, middle, and basal OHCs, double labeling experiments with antibodies against SK2 channel and BK channel demonstrate that SK2 channel and BK channel co-exist in the middle and basal OHCs [26,27]. By whole cell patch clamp recordings, it has been reported that BK channels play important roles in cholinergic inhibition of high frequency cochlear OHCs [26]. Then, after electrically stimulating the olivocochlear bundle in the BK^−/−^ ears, the OC-mediated suppression is reduced, but not eliminated at all frequencies. The remaining suppression is blocked by strychnine, suggesting involvement of α9/α10 cholinergic receptors, which are coupled to activation of the remaining SK2 channels. These results indicate that the efferent inhibition in the OHCs is combined action of BK and SK2 channels throughout the cochlea [27], but the subtype of cholinergic receptors mediating BK channels in OHCs is subtler.

According to the results that the efferent inhibition in the OHCs is combined action of BK and SK2 channels throughout the cochlea, it is reasonable to suppose that this efferent inhibition in the vestibule may involve both BK and SK2 channels. In the present study, we showed that VHCs II isolated by trypsin routinely displayed the α9nAChR sensitive SK2 current (Figure 2), but was insensitive to IBTX, a specific blocker of BK channels, while VHCs II isolated by collagenase IA routinely displayed the m2mAChR sensitive BK current (Figure 1), but was insensitive to apamin, a specific blocker of SK2 channels. According to these results, although the co-existence of BK and SK2 channels in VHCs II, we speculate that the efferent inhibition in the VHCs II might not be the combined action of these two channels. The reason may be as follow: we demonstrated that ACh activated BK currents were mediated by m2mAChR [19], while a recent study showed that m2mAChR was only present in olivocochlear neurons in cochlea, including their axons in the nerve trunk and the intraganglionic bundle and the osseous spiral lamina, as well as their terminals in the inner spiral bundle and underneath each of the three outer hair cell rows by immunohistochemistry. Besides, single cell RT-PCR also showed that m2mAChR was not present in OHCs [30]. ‘Knockout’ mice missing either α9 or α10 nAChRs failed to show suppression of distortion product otoacoustic emissions (DPOAEs)) during efferent fiber stimulation [31,32] and their hair cells no longer responded to local application of Ach [32]. Therefore, these results indicated that the subtypes of AChR mediating BK channel in cochlea may be α9/α10nAChR, which also mediated SK2 channel at the same time in OHCs. Nevertheless, our studies showed that BK channel mediated cholinergic inhibition in VHCs II by m2mAChR, which stimulated G_i_βγ-mediated excitation of AC/cAMP activities and led to the phosphorylation of Ca^2+^ channels, resulting in the influx of Ca^2+^ and opening of the BK channel [19]. Based on these kinetics features, due to the involvement of second messenger, the signal transduction of m2mAChR mediated BK channel would be activated slower than that of α9nAChR mediated SK2 channel. In spite of the coexistence of these two receptors on the VHCs II, ACh would more quickly activate the α9nAChR, which mediated a fast outward current SK2 and hyperpolarized membrane potential, thereby masking m2mAChR response in VHCs II.

#### 2.4.2. The Mechanisms Underlying the Changes of the ACh Response in Trypsined Cells after Exposure to Collagenase IA

In order to isolate individual vestibular hair cells from mammalian vestibular epithelium, the enzymes are commonly used, including papain and several serine proteases [33–36]. It has been reported that some collagenases have been successfully used to isolate cells from the cochlea [37–40], but they are rather ineffective in isolating mammalian VHCs II [16]. Nevertheless, our previous studies showed that ACh activated m2AChR mediated BK currents by using collagenase IA to isolate VHCs II, yet previous studies routinely demonstrated that ACh activated α9nAChR mediated SK2 currents by using trypsin to isolate mammalian VHCs II. This failure to demonstrate routine ACh responses led to speculation that the α9nAChR might undergo proteolysis during the collagenase IA isolation process and become non-functional. Indeed, there are several instances in the literature where enzymes have been shown to alter physiological responses of α9nAChR/SK2 complex. Armstrong and Roberts [22] have recently showed that papain altered particular ion channels in frog saccular hair cells including SK2, and Holt JC [16] has also recently showed that perfusions with either protease or papain permanently abolished the α9-nicotinic response in isolated saccular hair cells. According to these previous studies about the proteolytic effects on α9nAChR/SK complex, we speculate that it maybe collagenase IA that permanently abolished the α9-nicotinic response in trypsin isolated VHCs II. Therefore, we would record m2AChR mediated BK currents in collagenase IA isolated VHCs II.

Meanwhile, the possible mechanism of proteolysis of α9nAChR/SK2 complex by collagenase IA but not trypsin may be as follow:

Collagenase IA, a crude enzyme containing 10 to 18 enzymes, including two specific collagenases, clostripain, trypsin and a neutral protease caseinase (Sigma Product information), and most of them are proteases. it has been showed that perfusions with protease could permanently abolished the α9nAChR response in isolated saccular hair cells, while these inactivated enzymes became completely ineffective at abolishing the α9nAChR response [16]. Therefore, we speculated that the protease of clostripain and caseinase contained in crude collagenase IA may exert the proteolytic effect on the α9nAChR/SK2 complex as a result that clostripain specifically cleaves protein on the C-terminal of arginine residues [41] and caseinase has a broad range of amino acid specificity cleavage points, acting at the neutral pH.

However, trypsin is a polypeptide containing approximately 220 amino acid residues with 6 disulfide bridges and 1 calcium ion, which highly cleaves on the C-terminal side of lysine and arginine [42]. It has been reported that the voltage-sensitive Na^+^, K^+^ and Ca^2+^ channels which have been shown to contain trypsin-sensitive inhibitory domains share some common features: (1) large subunit molecular weights; (2) organization into domains containing six membrane spanning helices and a number of conserved motifs [43]. Besides, SK2 channel seems to belong to the trypsin-sensitive ion channel family due to the features as below [44]: (1) it contains 553–580 KD amino acids and 6 putative transmembrane domains; (2) there are five possible hydrolysis sites along its four extracellular loops. However, the arginine-glutamate bond in the third loop of SK2 channel should slow or impair the proteolytic effect since it has been reported that the neighboring acid residues such as aspartate and glutamate can impair the proteolytic effect of trypsin [45]. Therefore, in the present study, we showed that trypsin has little damaging effect on the function of α9nAChR in vestibular hair cells, which is in consistent with previous study [16].

Based on these reports, we speculated that the function of α9nAChR/SK complex may be irreversibly abolished after brief exposure to collagenase IA. Whether the lost activation of the SK2 current is a result of the proteolysis of SK2 alone or both α9nAChR and SK2 should be further investigated.

Meanwhile, as shown in Figure 3B, after exposing to collagenase IA, there was a downward shift in the baseline. It is well known that the resting membrane is mainly permeable to K^+^; thus, K^+^ channels play an important role in regulating the resting membrane potential, including Kir channel [46] and K_V_ channel [47]. Armstrong *et al.* showed that Kir channel [48,49] and K_V_ channel [22] were altered by enzymatic dissociation and K_V_ current [22] was apparently eliminated. Therefore, the downward shift in the baseline observed after the application of collagenase IA might be due to alteration of K^+^ channel by collagenase IA in VHCs II, which finally affects the resting membrane potential of the VHCs II.

## 3. Experimental Section

### 3.1. Animal Procedures and VHCs II Preparation

The Animal Care and Use Committee of Tongji Medical College approved these animal experiments. Briefly, 75 young guinea pigs (weighing 250–300 g, 6–10 weeks old) were deeply anesthetized by intramuscular injection with 0.3 mL of a mix of ⅓ xylazine (2%, Rompum, Bayer, German) and ⅔ ketamine hydrochlorate (50 mg/mL, Ketalar, Parke-Davis, France) and decapitated. Then, two methods of hair cells preparation involving different enzymes were applied independently.

### 3.2. VHCs II Isolated by Collagenase IA

Following decapitation, the vestibular epithelium was removed and incubated for 3 min at room temperature (20–24 °C) with 0.8 mg/mL collagenase IA in a low Ca^2+^ and Mg^2+^-free balanced salt solution (137 mM NaCl, 5.4 mM KCl, 0.1 mM CaCl_2_, 0.2 mM Na_2_HPO_4_, 0.4 mM KH_2_PO_4_, 10 mM glucose; pH = 7.2). Washing with normal external solution containing 2 mM CaCl2 stopped the enzymatic action. Finally, hair cells were mechanically dissociated by gentle trituration and were left to settle on the bottom of the experimental chamber coated with rat tail collagen.

### 3.3. VHCs II Isolated by Trypsin

Following decapitation, the vestibular epithelium was removed and incubated for 3 min at room temperature (20–24 °C) with 0.8 mg/mL trypsin in normal external solution as described below. Next, the normal external solution containing trypsin inhibitor was used to stop the enzymatic action. Finally, hair cells were mechanically dissociated by gentle trituration and were left to settle on the bottom of the experimental chamber coated with rat tail collagen.

### 3.4. Electrophysiology

VHCs II were readily identified by the cylindrical shape and absence of a distinct neck region [1]. The current activated by ACh was recorded in a whole-cell configuration, using an Axon-200B patch clamp amplifier (Axon Instruments, Foster City, CA, USA). Patch electrodes were pulled from borosilicate glass capillaries, using a Model P-97 electrode puller (Sutter Instrument, Novato, CA, USA). Electrode resistances ranged from 3 to 6 MΩ when filled with the internal solution, as described below. Records were low-pass filtered at 5 kHz with a four-pole Bessel filter. After gigaseal formation onto the basolateral membrane of VHCs and membrane disruption, the membrane capacitance was 4.6 ± 1.3 pF on average (*C*m, *n* = 6), and series resistance (*R*_S_, 6–15 MΩ) was compensated for up to 80%. The drug was applied to a patched cell at a holding potential of −50 mV.

The external solution component was as follow: 150 mM NaCl, 5 mM KCl, 2 mM CaCl_2_, 1 mM MgCl_2_, 5 mM glucose, and 10 mM HEPES (pH = 7.2). The internal solution component was as follow: 150 mM KCl, 2 mM MgCl_2_, 0.1 mM CaCl_2_, 3 mM Na_2_ATP, 5 mM EDTA and 10 mM HEPES (pH = 7.2).

The test solutions were applied to cells by a gravity-delivered linear barrel microperfusion system described elsewhere [18]. The microperfusion system consisted of a series of fused silica tubes (eight tubes with outer diameter of 150 μM and internal diameter of 100 μM) connected to a series of independent reservoirs. The tip of the tube was positioned approximately 100 to 150 μM from the cells. This microperfusion system was manipulated by shifting the tubes horizontally with a Leitz micromanipulator (ACS01, Leitz Corp., Wetzlar, Germany).

### 3.5. Drugs

All drugs were purchased from Sigma (St. Louis, MO, USA). Trypsin inhibitor (T9128), ACh (A6625), apamin (A9459), iberiotoxin (IBTX, I5904), methoctramine (M105) and strychnine (S7001) were directly dissolved in the external solution.

### 3.6. Data Analysis

Data were analyzed and plotted using the pCLAMP8.1 Clampfit 8.1 software (Axon Instruments, Foster City, CA, USA) and SigmaPlot 9.0 (Systat Software, Richmond, CA, USA). Results are presented as the mean ± SD, and statistical significance was determined using the t test. Differences were considered to be significant if *p* < 0.05; all differences listed were statistically significant, unless stated otherwise.

## 4. Conclusions

In conclusion, our findings provide evidences that BK channel and SK channel, as well as their corresponding m2mAChR and α9nAChR may co-exist in the same VHCs II of guinea pig, but the efferent inhibition in the VHCs II is not combined action of BK and SK2 channels as in cochlea. The physiological significance of m2mAChR mediated a slower BK current in VHCs II and the co-existence of these two ACh receptors sensitive currents in the same mammalian VHCs II should be further elaborated. Moreover, the alternative presence of these two ACh receptors sensitive currents is dependent on the isolating preparation with different enzymes. The proteolytic mechanisms of different enzymes on the α9nAChR/SK2 complex need further elucidation.
